# A Case Report of Neurofibroma of the Tongue Presented as a Solitary Lesion

**DOI:** 10.7759/cureus.65598

**Published:** 2024-07-28

**Authors:** Gowtham Narasimhan, Prasad T Deshmuk, Sagar S Gaurkar, Chandra Veer Singh, Farhat Q Khan

**Affiliations:** 1 Otolaryngology, Jawaharlal Nehru Medical College, Datta Meghe Institute of Higher Education and Research, Wardha, IND

**Keywords:** traumatic neurofibroma, tongue tumors, tongue lesions, tissue mass of tongue, benign tumors, oral cavity tumors, neurofibroma

## Abstract

Neurofibroma are rare occurrences in the oral cavity with the tongue as the most common location in the oral cavity being affected by neurofibroma. Neurofibroma are usually asymptomatic, irregular tissue masses of benign nature with a small rate of malignant conversion. Recurrence rates are also low in the neurofibromas of the oral cavity. It is rare in India with only a few cases reported to date. Hence, we report this case of a 63-year-old female with a tissue mass present on the right side of her tongue for the last five years, with a progressive nature. The mass was associated with pain during chewing food for the last three months. She was managed by a wide local incision and was reported well recovering at a three-month follow-up.

## Introduction

Neurofibroma is a type of benign tumor of the peripheral nerve sheath of the tongue reported to be arising from endoneurial fibroblasts, perineurial cells, and Schwann cells [1]. It is characterized by slow growth and a benign nature. This can be observed as a solitary growth or in the form of multiple lesions [1,2]. Clinically it is observed as firm, irregular, and non-ulcerative, which is commonly asymptomatic [2,3]. These tumors are not common in the oral cavity, usually reported in extra-oral locations [3-5]. The incidence of solitary neurofibroma in the oral cavity is reported as 6.5% [6]. A retrospective study of 20 years of data from central India reported a total of 14 such cases with a slight female predominance [7]. Radiological screening tools such as X-ray imaging, computed tomography (CT), and magnetic resonance imaging (MRI) can help establish the diagnosis, which can be confirmed after histopathological analysis [1,3]. Its conversion to malignant forms is reported between 3%-15% and surgical excision remains the mainstay treatment with advice for regular follow-up [1,4,5,7]. We present a case of a 63-year-old female with a long history of a tissue mass on her tongue without any pain or associated co-morbidities.

## Case presentation

**Figure 1:**
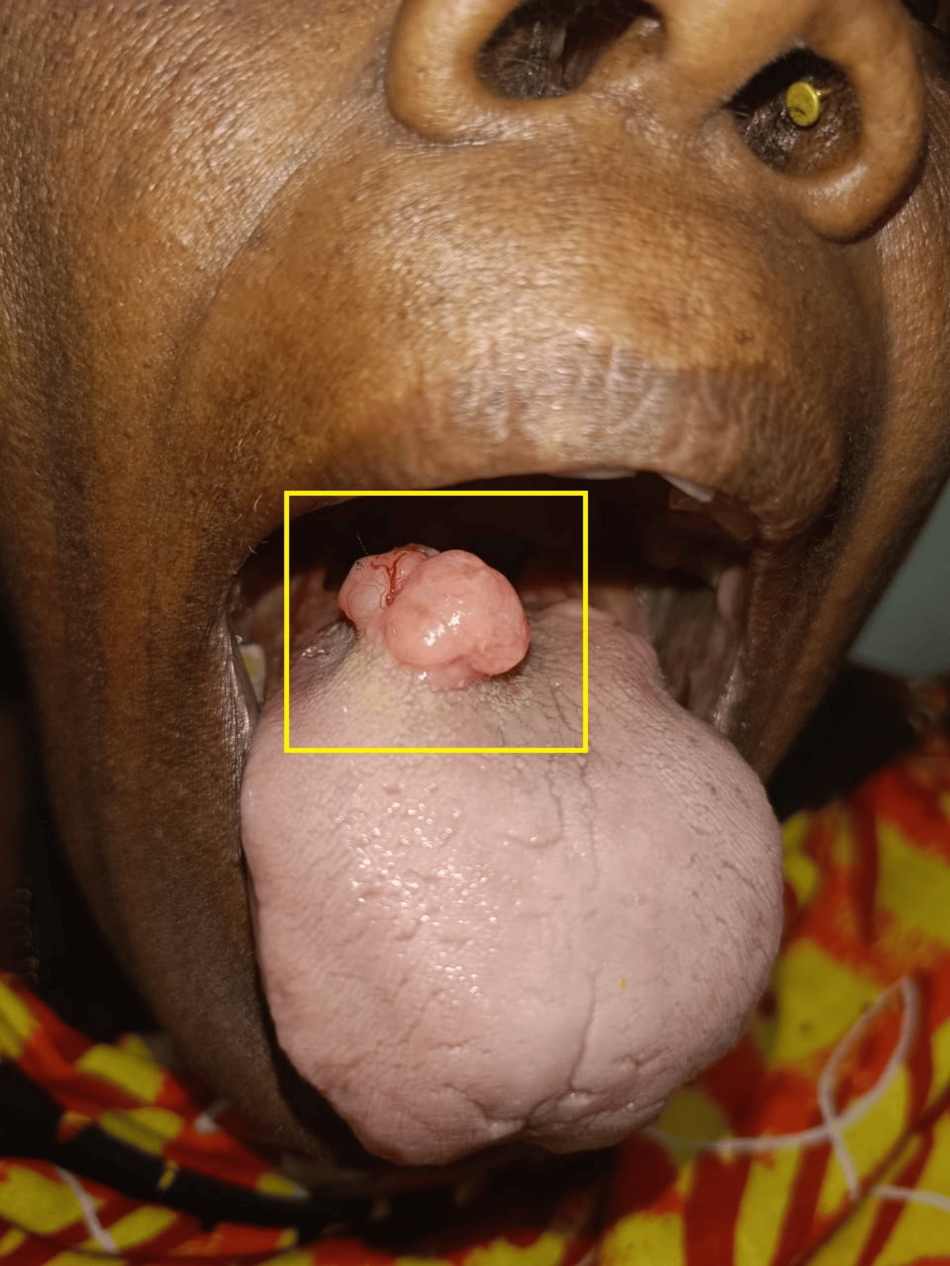
Physical presentation of the mass of the tongue

A 63-year-old female visited our hospital with a major complaint of a tissue mass over the dorsum of the tongue for five years. The mass was observed as painless oval-shaped swelling at the right side between the anterior 2/3rd and posterior 1/3rd of the dorsum of the tongue with gradual progression of size associated with a burning sensation. This was associated with pain during chewing of the food for the last three months. On examination, approximately 3.0 cm x 2.0 cm, oval-shaped swelling was observed at the right side of the dorsum of the tongue, slightly tender and firm consistency. It was noted to have an irregular surface and was non-ulcerative (Figure 1).

**Figure 2:**
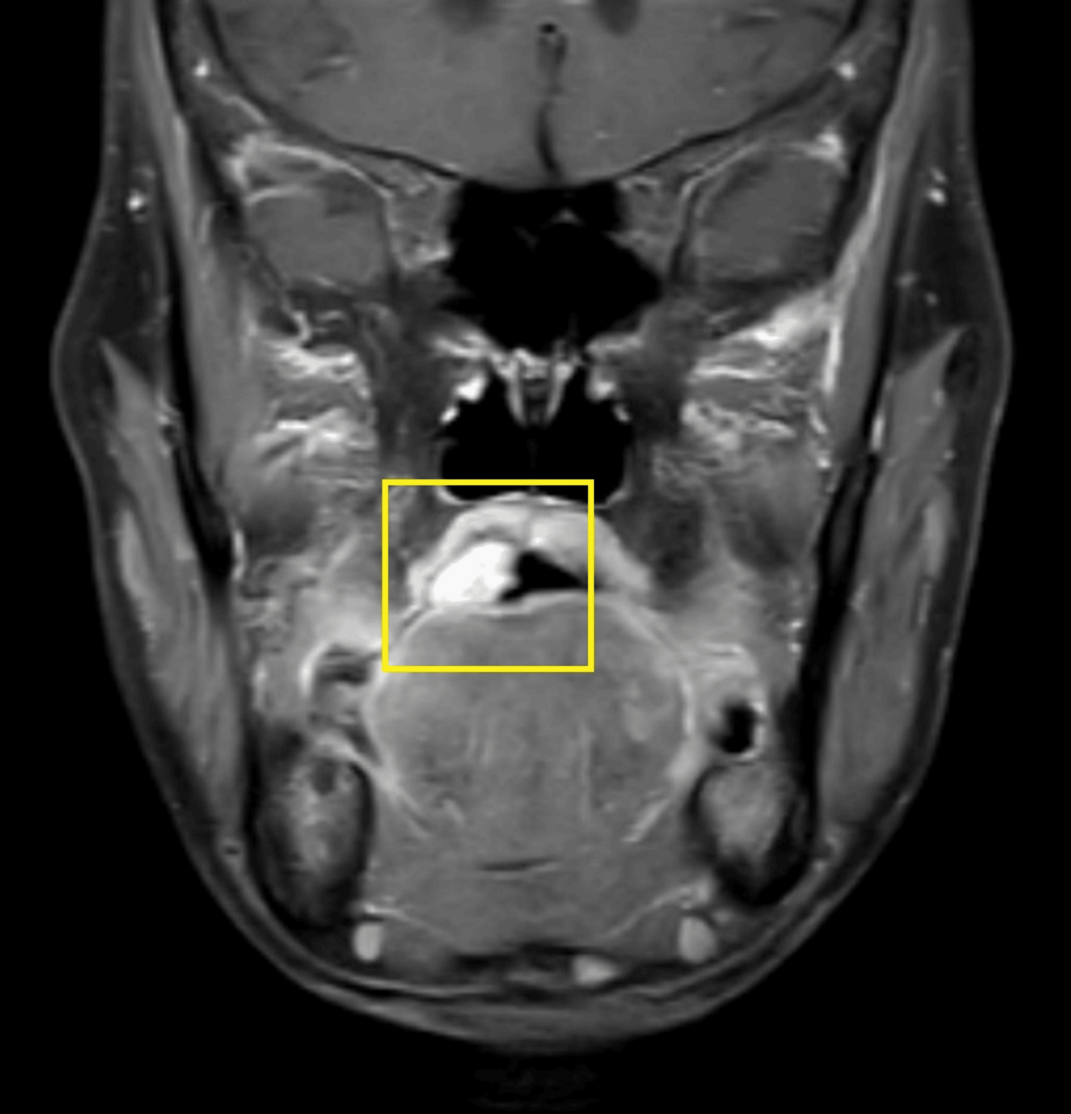
MRI of the oral cavity (coronal view) MRI: magnetic resonance imaging

**Figure 3:**
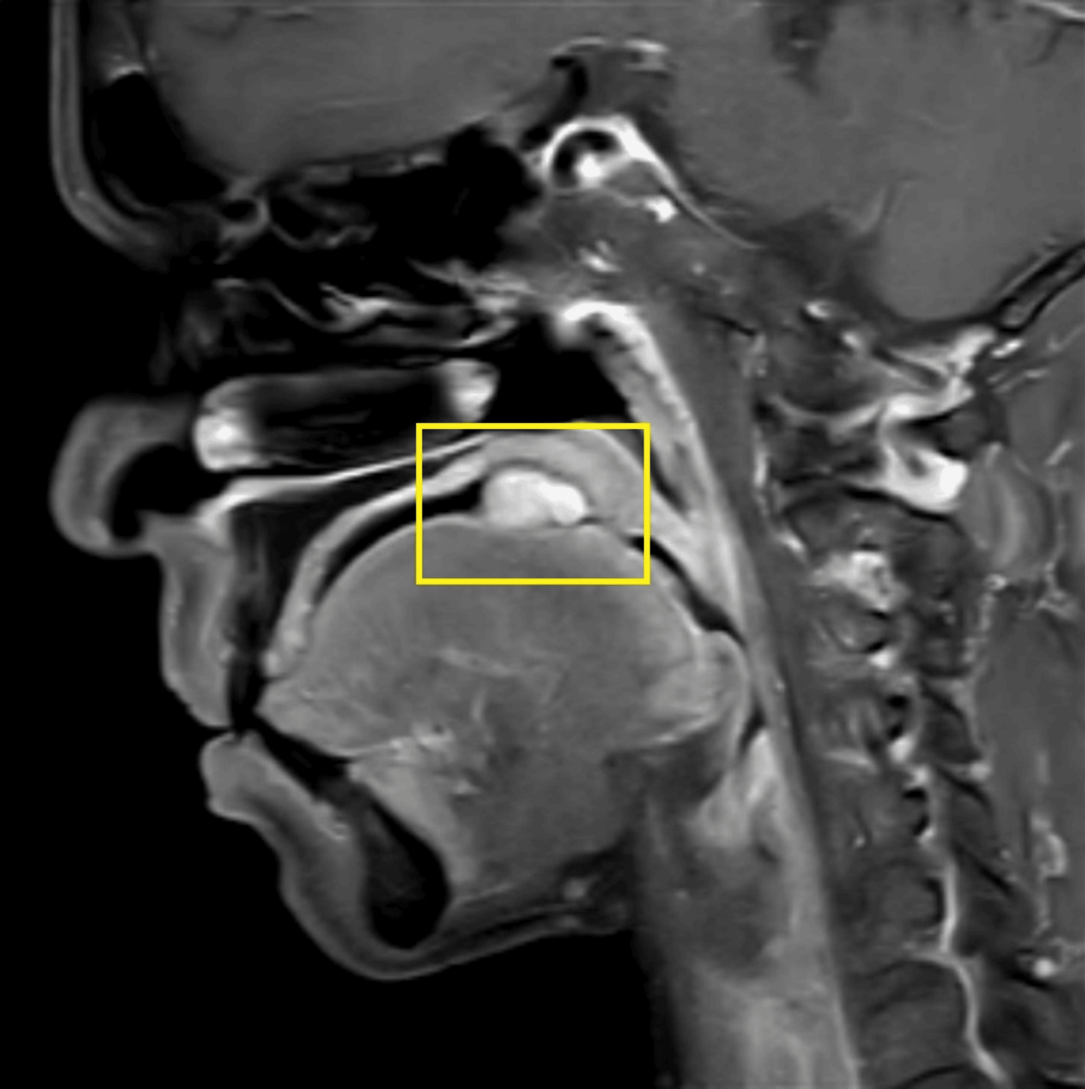
MRI of the oral cavity (sagittal view) MRI: magnetic resonance imaging

**Figure 4:**
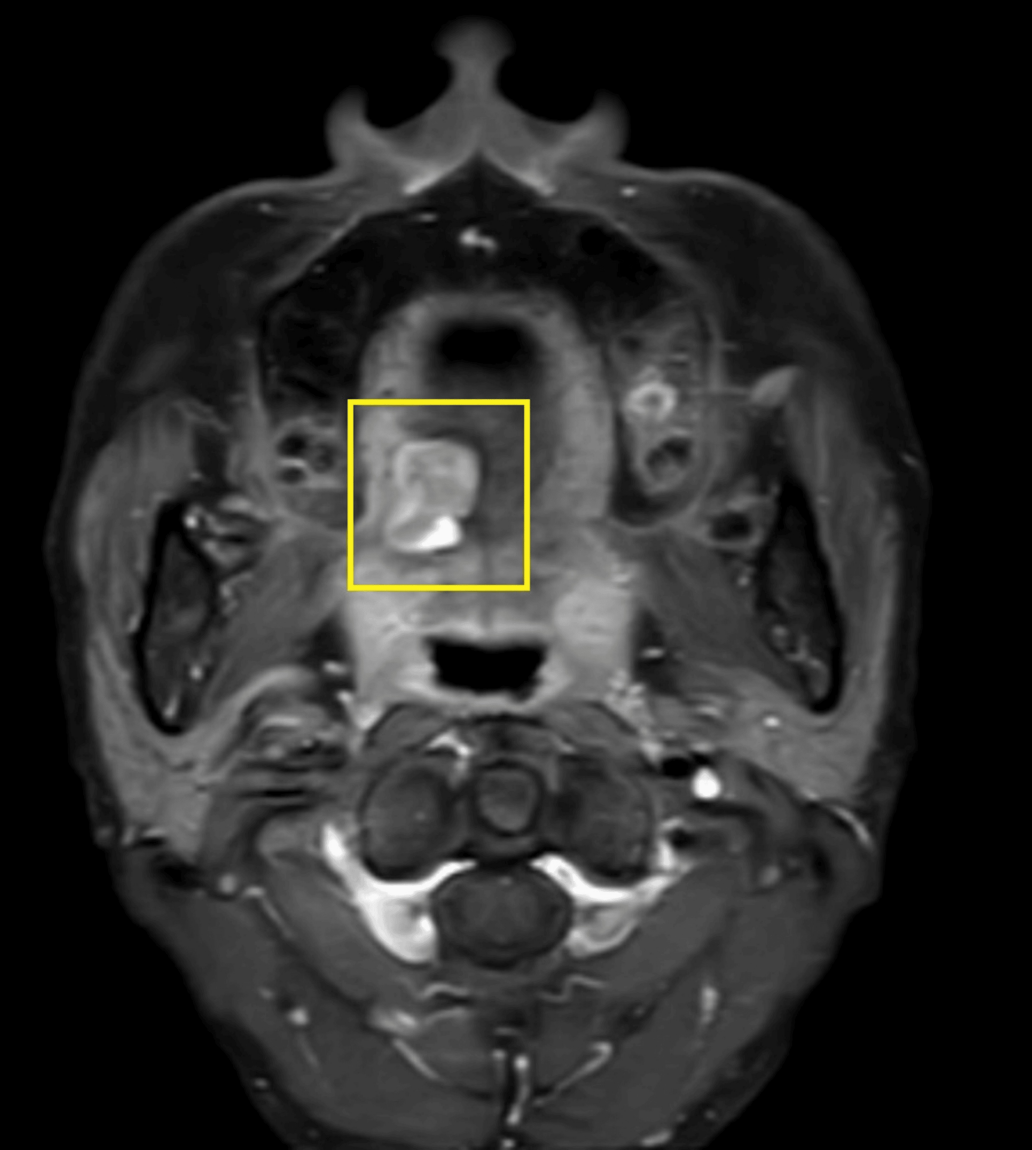
MRI of the oral cavity (axial view) MRI: magnetic resonance imaging

A general physical examination showed a moderately built female with normal vital signs. There were no signs of clubbing, anemia, and cyanosis. No significant extra-oral manifestations. There was no palpable cervical lymphadenopathy or other local lymphadenopathy. Physical examination showed no similar lesions observed in any other part of the patient’s body. There was no skin pigmentation, hearing issues, or any evidence, which was suggestive of any associated systemic disorder or family history. An MRI of the oral cavity with the neck showed an altered signal intensity lesion measuring approximately 3 cm x 2.4 cm, involving the right side of the base of the tongue. There was no invasion observed in the floor of the mouth and extrinsic muscles of the tongue with no bony invasion seen. Lymph nodes were found to be normal in the bilateral cervical region. It appeared hyper-intense on short tau inversion recovery (STIR) and showed moderate enhancement in the post-contrast study (Figures 2, 3, 4).

**Figure 5:**
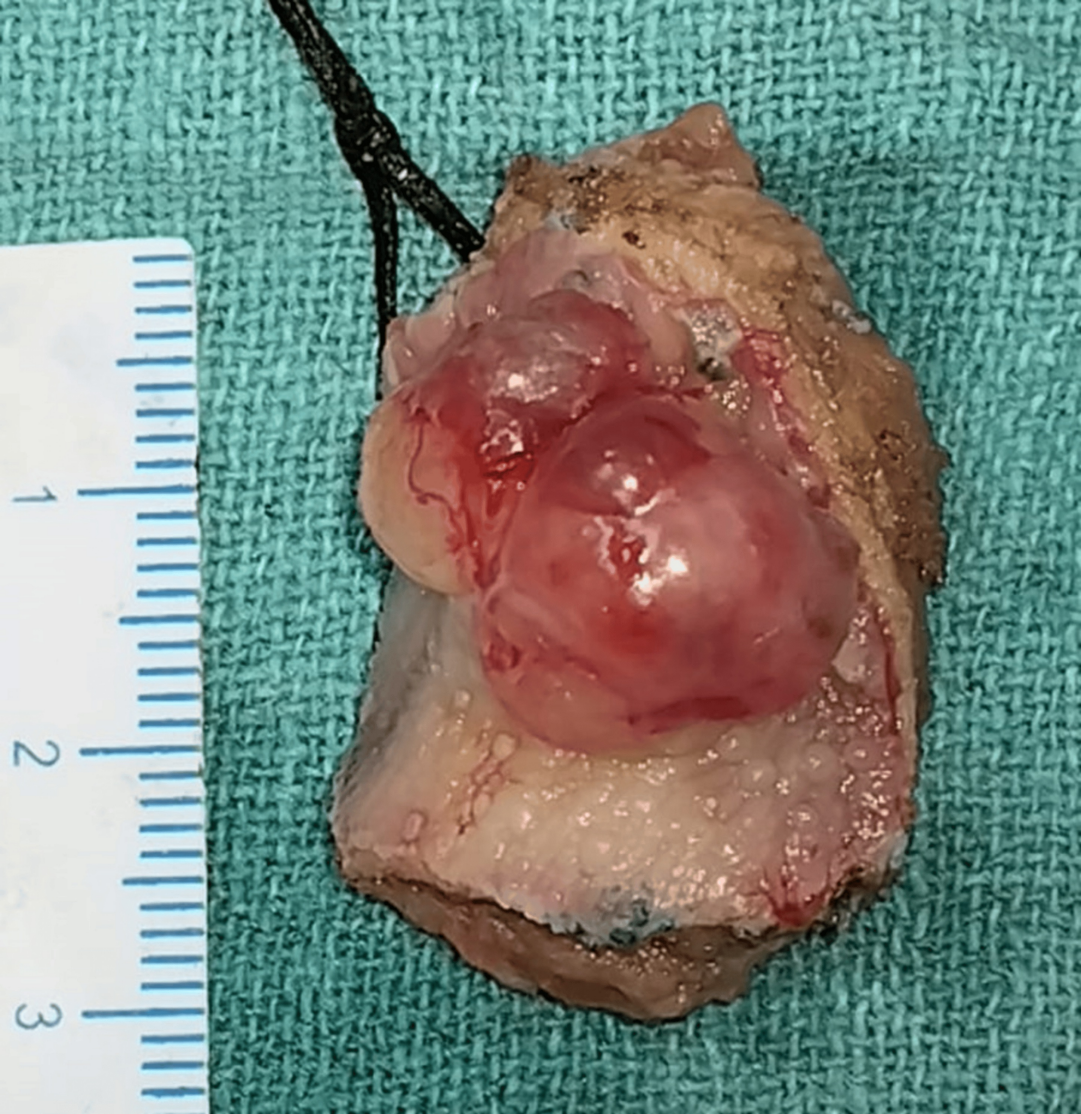
Excised specimen

**Figure 6:**
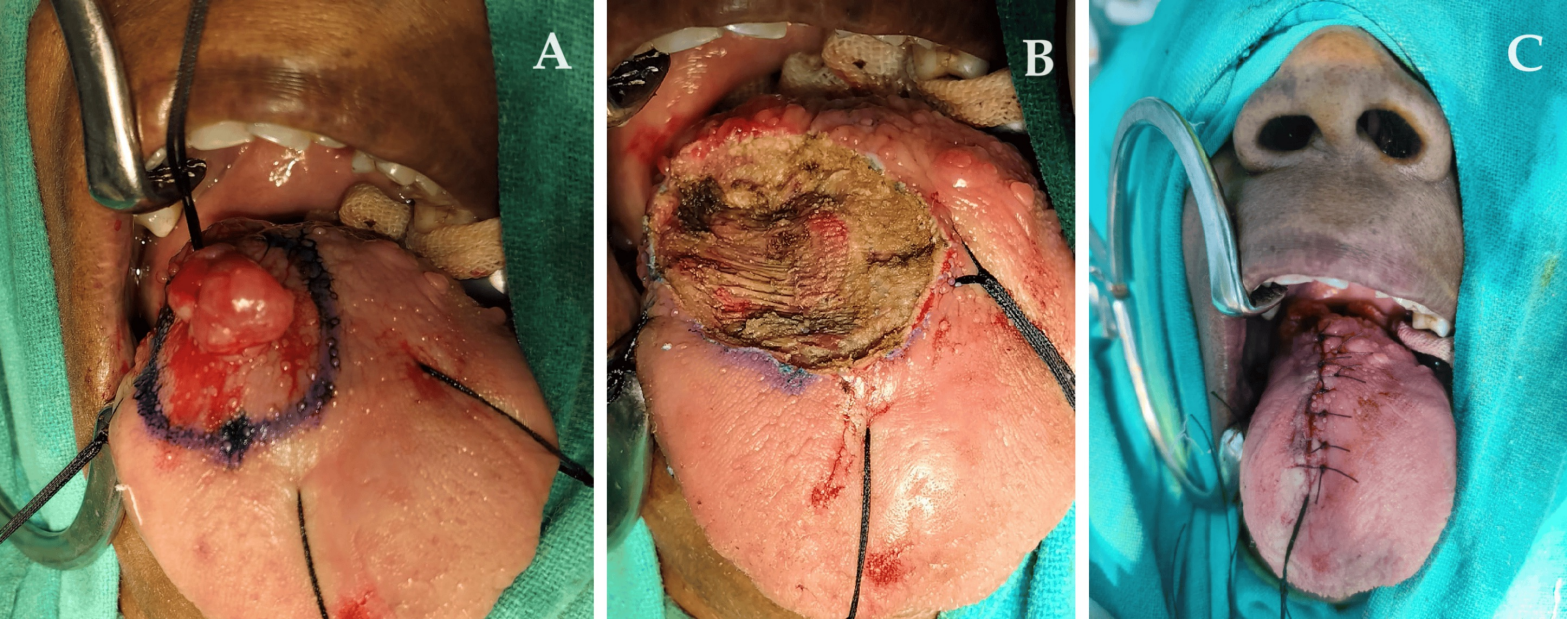
Intra-operative images of the procedure (A) shows pre-excision, (B) shows immediately after excision of the tumor, and (C) shows a post-operative image.

The patient underwent an excisional biopsy of the tongue circumferential with a 1 cm margin. The tumor was excised and closed with interrupted sutures (Figures 5, 6).

**Figure 7:**
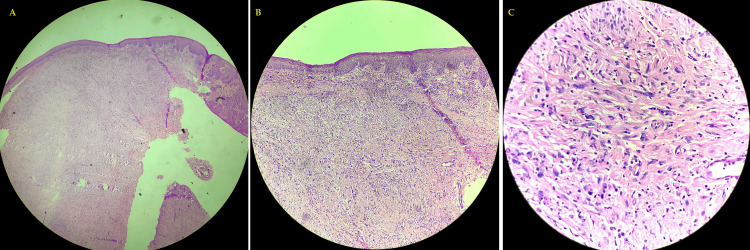
Histopathological study of the excised specimen (A) scanner view of hematoxylin and eosin-stained slide show hypertrophied squamous epithelial lining with cells arranged in sheets. (B) 10X magnification of hematoxylin and eosin-stained slide show the dermal invasion of spindle cells arranged in sheets along with fibro-collagenous tissue. (C) 40X magnification of hematoxylin and eosin-stained slide show dermal invasion of spindle cells arranged in sheets along with fibro-collagenous tissue.

The excised specimen was sent for histopathological examination, which showed hypertrophied squamous epithelial lining with moderate dysplasia. Deeper tissue showed fibro-collagenous tissue and congested blood vessels with mild information. These features were suggestive of traumatic neurofibroma (Figure 7).

The patient was followed up at three months post-surgery with no fresh complaints. She was able to eat without any pain and speak well.

## Discussion

Neurofibroma is infrequent in the head and neck region. In India, neurofibroma of the oral cavity is a rarity. There are few cases of solitary neurofibroma reported to date with a slight female preponderance observed in the published literature from India [7]. These neurofibroma of the oral cavity are further categorized into two types: neurofibromatosis (NF) type 1 and NF type 2 [2]. The total incidence of neurofibroma occurring in the head and neck region is noted as 25%, out of which 6.5% have been observed to occur in the oral cavity [2,6,8]. NF type 1 and NF type 2 are associated with mutations of NF1 and NF2 gene, respectively. NF type 1 is usually observed with multiple skin lesions, skin discoloration, and optic nerve-related tumors, whereas NF type 2 is observed as schwannomas and meningiomas. The incidence rate of NF type 1 is observed as one in 3000 births irrespective of ethnicity [9]. The tongue is reported as a common site for the neurofibromas of the oral cavity, followed by the palate, mandibular ridge, maxillary ridge, mucosa, lip, intra-bony mandibular, and gingiva [4-7]. There were similar observations of solitary neurofibroma in the case report by Mahmud et al. and Iyer et al. in 73- and 34-year-old females, respectively [1,4]. The case reported by Iyer et al. had a nearly similar presentation with a long history without any pain, though the size of the tumor was small compared to this case [4]. Neurofibroma are uncommon in the oral cavity with very few cases reported from India. Commonly the neurofibromas of the oral cavity are rarely presented as solitary lesions; they are usually categorized as either NF type 1 or NF type 2 based on the locations of associated lesions. These are widely categorized into plexiform neurofibromas and cutaneous neurofibromas [9,10]. This case was presented as a long asymptomatic solitary lesion in the tongue as opposed to other cases [2,4,5]. The diagnosis was confirmed by MRI imaging, which was affirmed after the histopathological study of the excised sample. It is crucial to report more cases of fibroma of the oral cavity as, these tumors are benign and asymptomatic, most often ignored by the patients raising the chances of acute presentations and conversion to malignant forms. Surgical excision is the most preferred treatment option for these tumors with a regular follow-up to keep a check on recurrence.

## Conclusions

Neurofibroma of the oral cavity can be a clinical challenge both due to its rarity and the probability of ignorance from the patients with any asymptomatic masses, as in this case, which in turn increases the risk of metastasis. It is important to address any mass of progressive nature. Prompt diagnosis and timely treatment are required, with the advice of periodic follow-up in scrutinizing recurrence, if any. 
